# Supplementary material for: Fundamental frequency sequence amplitude estimator for power and energy applications
Source: PLoS One. 2022 Aug 1;17(8):e0270851. doi: 10.1371/journal.pone.0270851 (PMC9342759; doi:10.1371/journal.pone.0270851)
Supplement: S1 File — (PDF) [file pone.0270851.s001.pdf]

Fundamental Frequency Sequence Amplitude Estimator for Power and Energy Applications

Hafiz Ahmed[1*], Zoheir Tir[2], Samet Biricik[3], Mohamed Benbouzid[4,5]

1 Nuclear Futures Institute, Bangor University, Bangor, Gwynedd UK LL57 2DG, UK
2 LEVRES Laboratory, University of El Oued, 39000 El Oued, Algeria
3 Department of Electrical and Electronic Engineering, European University of Lefke, Lefke 99728, Turkey
4 University of Brest, UMR CNRS 6027 IRDL, 29238 Brest, France
5 Logistics Engineering College, Shanghai Maritime University, Shanghai 201306, China

* hafiz.ahmed@bangor.ac.uk; hafiz.h.ahmed@ieee.org

Abstract

A grid-synchronization-based fundamental frequency positive-sequence (FFPS) and negative-sequence (FFNS) amplitudes estimation technique is proposed for unbalanced and distorted grid. In this technique, the sequence amplitudes are extracted by extracting the phase-angle of the FFPS and FFNS components. The extracted phase-angles have DC and double frequency AC components. The AC component is filtered out by using a Moving Average Filter (MAF) of appropriate window length. From the extracted phase-angle, the unknown frequency can be estimated by using a suitable controller. A frequency-fixed equidistant samples-based pre-loop filter is also applied to eliminate the effect of measurement offset. The proposed technique has a very simple structure and is easy to tune. Small-signal modeling-based stability analysis and gain tuning procedure are also provided. The proposed technique strikes a good balance between fast convergence and disturbance rejection capability. Comparative numerical simulation and experimental results with similar other techniques demonstrate the suitability and performance enhancement by the proposed technique.

Introduction

Grid-synchronization plays a vital role in the control of grid-connected power electronic systems. Synchronized or in-phase operation of the power electronic systems with the grid plays an important role in enhancing the overall operational efficiency of the system. Grid-synchronization technique extracts the parameters of the grid voltage signals which are then used inside the controller of grid-connected converters [1–7].

Owing to the importance of grid-synchronization in control and monitoring of grid-connected converters, several techniques are already proposed in the literature. Zero-crossing detection [8] is one of the pioneering technique in this area. By detecting the transition of the signal from negative to positive, this technique can estimate the frequency in real-time. Various power quality disturbances can adversely affect the performance. To overcome these limitations, this technique has been enhanced with Kalman filter in [9]. In this approach, a Kalman filter is used to generate quadrature signals and then zero-crossing detection is applied to the signals for frequency estimation. However, this technique can be sensitive to DC offset which is often caused by the current transformer saturation.

In steady-state operation, the power grid is quasi-periodic with known nominal period. This makes discrete Fourier transform type techniques [10] a very suitable tool for grid-synchronization application. As large number of samples need to be stored, this type of techniques typically have large memory and consequently high processing power requirement. This can be problematic for low-cost real-time hardware. In the off-nominal case, the performance can be improved by using variable sampling operation. Output filters of converters are typically designed by assuming a fixed sampling frequency condition. As a result, variable sampling frequency operation can be problematic for the overall converter control system.

Using simple trigonometric manipulation, the grid voltage signals can be easily modeled into parametric form with time-varying regressors. This enabled the application of least-square type algorithms [11, 12] for grid-synchronization applications. In the nominal condition, the regressor matrix size is low. However, in the off-nominal case, the regressor matrix can be very large. This necessitates storing a large number of samples. Moreover, real-time computation of large number of samples can be highly computationally demanding. Least-square techniques can handle time-varying nature of the grid voltage parameters by using variable forgetting factor or covariance resetting mechanism. Kalman filter [11] can directly tackle this problem without using any forgetting factor or resetting mechanism. Kalman filter has excellent noise robustness property, however, its tuning requires accurate information of the process and measurement noise covariance properties. This may not be always easily available. Recently, it has been shown experimentally in [13] that least-square technique with integral of the estimation error cost-function achieves better noise robustness than Kalman filter. Linear observer [14] can provide fast detection, however, has a sensitivity to unmodeled dynamics such as harmonics.

Phase-Locked Loop (PLL) [15–18] is another technique that is extremely popular in the literature. Out of various PLL techniques available in the literature, Synchronous Reference Frame (SRF)-PLL [15] is undoubtedly the most used one. SRF-PLL uses the Park transformation the phase detector and the output is passed through a low-pass filter to extract the unknown grid frequency. In the balanced grid case, SRF-PLL has excellent dynamic and steady-state performances. However, the performance deteriorates in the presence of disturbances such as unbalance, harmonics, etc. To overcome the limitations of SRF-PLL, one potential solution is to use additional band-pass filters such as self-tuning filter [19], second-order generalized integrator [20], adaptive notch filter (ANF) [21], etc. These filters are typically applied in the stationary reference frame to extract the Fundamental Frequency Positive Sequence (FFPS) component. SRF-PLL then extracts the unknown frequency from the FFPS component. In the presence of band-pass filters, the tuning of PLL can be complicated. Unlike the band-pass filters, Moving Average Filter (MAF) is typically employed in the synchronous reference frame [22] to mitigate the negative effect of unbalance. Depending on the window length of the MAF, MAF-PLL can completely eliminate the harmonics and DC offset. However, MAF-PLL has a relatively slow dynamic response.

PLL's phase detector works in a closed-loop fashion. Unlike PLL, demodulation technique's phase detector works in an open-loop manner [23]. In demodulation, undesired high frequency component of the phase detector output is filtered through a low-pass filter. For effective cancellation of undesired components, the cut-off frequency of the filter needs to be high. This reduces the overall bandwidth of demodulation resulting in a slow dynamic response. To overcome this limitation, frequency-adaptive demodulation is proposed in [24]. This increases the bandwidth, however, it is only applicable for single-phase systems. Authors in [24] used three single-phase demodulation blocks to tackle three-phase system, however, this is not a constructive way to deal with a three-phase system as the computational burden is high. Recently, high bandwidth demodulation

technique was proposed in [25], however, it is not capable of handling unbalance. Similar to classical demodulation, many other open-loop grid-synchronization techniques are available in the literature [26, 27]. Open-loop techniques have fast convergence and unconditional stability. However, to achieve exact-synchronization, the frequency is often fed back to the filtering part of the open-loop technique. This results in a quasi open-loop structure for the overall system. As a result, unconditional stability property is no longer valid.

From the discussions so far, it is evident that there is a demand of grid-synchronization technique that can work under an unbalanced and distorted grid with potential DC offset rejection capability. This work, therefore, focuses on developing such a technique. For this purpose, this work proposes an enhanced phase detector inspired by the quasi type-1 PLL (QT1-PLL) [28, 29] that can extract both the FFPS and FFNS phase-angle using extended Park-type transformation. The extracted phase-angles have a double frequency component. This can be removed either by using a low-pass filter or a moving-average filter. The advantage of using a moving-average filter is that it can completely eliminate even-order harmonics for a suitably selected window length. The filtered FFPS/FFNS phase-angle can be used to extract the unknown grid frequency by passing it through an appropriate proportional controller. This choice of controller differentiates the proposed solution from that of PLL and FLL where proportional-integral and integral controllers are used, respectively. Finally, equidistant samples-based DC offset rejection Small-signal modeling-based tuning is proposed for the developed PLL. The proposed technique has a simple structure and is easy to implement. It can extract both FFPS and FFNS components without using any complicated filtering techniques such as orthogonal signal generator [30, 31] and/or cross-coupling observer/filter [17]. Moreover, it also doesn't require any parallel structure of filter/observer-bank for harmonic robustness [32]. This makes the proposed technique very simple to implement in low-cost embedded devices. The proposed method has a very simple gain tuning procedure as it has only one tunable gain. To facilitate the tuning, a small-signal model is developed that can be used for phase margin-based tuning. Moreover, by using same phase margin-based fair gain tuning, we have showed that the proposed PLL strikes a good balance between fast convergence and disturbance rejection capability compared to similar other techniques.

The proposed technique uses MAF to filter out the high frequency component. Many other PLLs use a similar approach such as MAF-PLL [33], QT1-PLL [28], Total QT1-PLL (TQT1-PLL) [22], MAF-based sequence estimator [34], DDSRF-PLL [17, Figure 19] with in-loop MAF, etc. Standard MAF and QT1-PLL can extract only FFPS component whereas the proposed technique can estimate both FFPS and FFNS amplitudes. TQT1-PLL has a similar sequence extraction capability like the proposed technique, however, it is very sensitive to sub- and inter-harmonics due to the use of reduced window length DSC block for sequence separation. *A priori* knowledge of harmonics is required for designing the MAFs in [34], however, no such knowledge is required for the proposed technique. DDSRF-PLL and the proposed technique has similar rotational speed but the proposed technique has a simple decoupling structure. Unlike DDSRF-PLL, no cross-coupling of the filtered variables are used which makes the proposed technique suitable for low sampling frequency operation [35]. Moreover, the proposed technique has a fast dynamic response thanks to proportional controller-based loop filter as opposed to proportional-integral-type loop-filter in DDSRF-PLL. These features differentiate the proposed method with respect to the relevant existing literature.

Development of the Proposed PLL

In the stationary reference frame (α, β), the unbalanced three-phase grid voltages can be written as:

$$V_\alpha = v_{\alpha 0} + v^+ \cos(\theta^+) + v^- \cos(\theta^-), \tag{1a}$$
$$V_\beta = v_{\beta 0} + v^+ \sin(\theta^+) - v^- \sin(\theta^-), \tag{1b}$$

where the superscript $+/-$ denotes positive/negative sequence, measurement offsets are denoted by $v_{\alpha 0}$ and $v_{\beta 0}$, and $\theta = \omega t + \phi$, v, ω and ϕ represent the amplitude, angular frequency and phase angle respectively. Nominal value of the angular frequency is denoted by ω_n. In this work, grid-synchronization-based FFPS and FFNS amplitudes estimation are considered. For this purpose, amplitudes of the FFPS and FFNS components, i.e., v^+ and v^- need to be extracted from the measured voltages V_α and V_β.

FFPS and FFNS Phase Angle Detector

In this Section, first, we assume that unbiased measurements are available, i.e., no DC offset in eq. (1). Elimination of the offset will be considered later on. Let us consider the following auxiliary signals:

$$SC = \sin(\hat{\omega} t) + \cos(\hat{\omega} t), \tag{2a}$$
$$CS = \cos(\hat{\omega} t) - \sin(\hat{\omega} t), \tag{2b}$$

where $\hat{\omega}$ is the estimated frequency. With respect to the auxiliary signals (2a) and (2b), let us consider the following transformation:

$$\begin{bmatrix} v_s^+ \\ v_c^+ \\ v_s^- \\ v_c^- \end{bmatrix} = \underbrace{\frac{1}{2} \begin{bmatrix} 1 & 1 & 1 & 1 \\ -1 & 1 & 1 & -1 \\ 1 & 1 & -1 & -1 \\ -1 & 1 & -1 & 1 \end{bmatrix} \begin{bmatrix} -SC & 0 \\ CS & 0 \\ 0 & SC \\ 0 & CS \end{bmatrix}}_{\mathcal{T}^m} \begin{bmatrix} V_\alpha \\ V_\beta \end{bmatrix}. \tag{3}$$

Similar to the conventional Park transformation-based phase detector, eq. (3) serves as the phase detector for the proposed approach. For further development in this section, let us assume that the PLL is in quasi-locked condition i.e. $\omega \approx \hat{\omega}$. To explain the operation of the proposed phase detector, let us consider the following signals obtained by multiplying the second and third matrices in the r.h.s. of eq. (3):

$$\begin{aligned} V_\alpha^1 &= -V_\alpha V_{sc}, \\ &= \frac{V^+}{2}\left[\sin\left\{(\omega-\hat{\omega})\,t+\phi^+\right\} - \sin\left\{(\omega+\hat{\omega})\,t+\phi^+\right\} - \cos\left\{(\omega+\hat{\omega})\,t+\phi^+\right\}\right] + \\ &\quad \frac{V^-}{2}\left[\sin\left\{(\omega-\hat{\omega})\,t+\phi^-\right\} - \sin\left\{(\omega+\hat{\omega})\,t+\phi^-\right\} - \cos\left\{(\omega+\hat{\omega})\,t+\phi^-\right\}\right] - \\ &\quad \left(\frac{V^+}{2}+\frac{V^-}{2}\right)\cos\left\{(\omega-\hat{\omega})\,t+\phi^+\right\}. \end{aligned} \tag{4a}$$

$$\begin{aligned} V_\alpha^2 &= V_\alpha V_{cs}, \\ &= \frac{V^+}{2}\left[\sin\left\{(\omega-\hat{\omega})\,t+\phi^+\right\} - \sin\left\{(\omega+\hat{\omega})\,t+\phi^+\right\} + \cos\left\{(\omega-\hat{\omega})\,t+\phi^+\right\}\right] + \\ &\quad \frac{V^-}{2}\left[\sin\left\{(\omega-\hat{\omega})\,t+\phi^-\right\} - \sin\left\{(\omega+\hat{\omega})\,t+\phi^-\right\} + \cos\left\{(\omega-\hat{\omega})\,t+\phi^-\right\}\right] + \\ &\quad \left(\frac{V^+}{2}+\frac{V^-}{2}\right)\cos\left\{(\omega+\hat{\omega})\,t+\phi^+\right\}. \end{aligned} \tag{4b}$$

$$
\begin{aligned}
V_\beta^1 &= V_\beta V_{sc}, \\
&= \frac{V^+}{2}\left[\sin\left\{(\omega+\hat{\omega})\,t+\phi^+\right\}+\sin\left\{(\omega-\hat{\omega})\,t+\phi^+\right\}+\cos\left\{(\omega-\hat{\omega})\,t+\phi^+\right\}\right]- \\
&\quad \frac{V^-}{2}\left[\sin\left\{(\omega+\hat{\omega})\,t+\phi^+\right\}+\sin\left\{(\omega-\hat{\omega})\,t+\phi^+\right\}+\cos\left\{(\omega-\hat{\omega})\,t+\phi^+\right\}\right]- \\
&\quad \left(\frac{V^+}{2}-\frac{V^-}{2}\right)\cos\left\{(\omega+\hat{\omega})\,t+\phi^+\right\}. \qquad (4c) \\
V_\beta^2 &= V_\beta V_{cs}, \\
&= \frac{V^+}{2}\left[\sin\left\{(\omega-\hat{\omega})\,t+\phi^+\right\}+\sin\left\{(\omega+\hat{\omega})\,t+\phi^+\right\}+\cos\left\{(\omega+\hat{\omega})\,t+\phi^+\right\}\right]- \\
&\quad \frac{V^-}{2}\left[\sin\left\{(\omega-\hat{\omega})\,t+\phi^-\right\}+\sin\left\{(\omega+\hat{\omega})\,t+\phi^-\right\}+\cos\left\{(\omega+\hat{\omega})\,t+\phi^-\right\}\right]- \\
&\quad \left(\frac{V^+}{2}-\frac{V^-}{2}\right)\cos\left\{(\omega-\hat{\omega})\,t+\phi^+\right\}. \qquad (4d)
\end{aligned}
$$

From the signals (4a)-(4d) and by considering $\omega-\hat{\omega}\approx 0$ and $\omega+\hat{\omega}\approx 2\hat{\omega}$, the following signals can be obtained by simple algebraic manipulation which is given in the first matrix in the right hand side of eq. (3):

$$
\begin{aligned}
v_s^+ &= \frac{1}{2}\left\{\left(V_\alpha^1+V_\beta^2\right)+\left(V_\beta^1+V_\alpha^2\right)\right\}, \\
&\approx V^+\sin(\phi^+)-V^-\sin(2\hat{\omega}t+\phi^-). \qquad (5a) \\
v_c^+ &= \frac{1}{2}\left\{\left(V_\beta^1+V_\alpha^2\right)-\left(V_\alpha^1+V_\beta^2\right)\right\}, \\
&\approx V^+\cos(\phi^+)+V^-\cos(2\hat{\omega}t+\phi^-). \qquad (5b) \\
v_s^- &= \frac{1}{2}\left\{\left(V_\alpha^1-V_\beta^2\right)+\left(V_\alpha^2-V_\beta^1\right)\right\}, \\
&\approx V^-\sin\left(\phi^-\right)-V^+\sin\left(2\hat{\omega}t+\phi^+\right). \qquad (5c) \\
v_c^- &= \frac{1}{2}\left\{\left(V_\beta^2-V_\alpha^1\right)+\left(V_\alpha^2-V_\beta^1\right)\right\}, \\
&\approx V^-\cos\left(\phi^-\right)+V^+\cos\left(2\hat{\omega}t+\phi^+\right). \qquad (5d)
\end{aligned}
$$

From signals (5a)-(5d), it is clear that each signal is composed of a DC component together with a double frequency component. The double frequency components can be removed by a moving average filter (MAF) with appropriate window length. In obtaining eq. (5a)- (5d), odd-harmonics are not considered. If considered, then, odd-harmonics will appear as double the fundamental frequency AC harmonic components in eq. (5a)-(5d). An advantage of MAF is that it can remove double the fundamental frequency harmonic AC components from eq. (5a)-(5d) if the window length is selected as $T_w = 0.5T$, where T is the signal period. Hence, MAF is selected in this work to enhance the harmonic performance improvement. The transfer functions of the continuous-time and discrete-time MAF are given by:

$$G_f(s) = \frac{1-e^{-T_w s}}{T_w s}, \qquad (6a)$$

$$G_f(z) = \frac{1}{N}\frac{1-z^{-N}}{1-z^{-1}}, \qquad (6b)$$

where N is the number of samples inside the window T_w. By passing the signals (5a)-(5d)

through the MAF (6b), the DC signals (7)-(10) are obtained:

$$v_s^{f+} \approx V^+ \sin(\phi^+), \tag{7}$$
$$v_c^{f+} \approx V^+ \cos(\phi^+), \tag{8}$$
$$v_s^{f-} \approx V^- \sin(\phi^-), \tag{9}$$
$$v_c^{f-} \approx V^- \cos(\phi^-). \tag{10}$$

These DC signals can be used for the PLL implementation.

Pre-Loop Frequency-Fixed DC Offset Rejection

In developing the proposed phase detector, no DC offset has been assumed. However, this may not be the case in practice due to various reasons. So, eliminating the DC offset is considered in this Section. In the PLL literature, several solutions are proposed to eliminate the DC offset. Out of them, some popular choices are based on additional integrator [36], low-pass filter [37], delayed signal cancellation [22, 30] etc. Out of these choices, DSC is a particularly suitable solution as it can be implemented as a pre-loop filter and has no tuning gain, thereby reducing the tuning simplicity unlike LPF or additional integrator-based solutions. As such, this approach has been adopted here. In the conventional DSC-based DC offset rejection approach, half-cycle delayed signals are used. Recently, in [31, 38, 39], an alternative approach has been presented where DC offset rejection can be done using an arbitrary length of delay. As such, the modified DSC approach is adopted in this work. For this purpose, let us consider two successively delayed samples of the Clarke-transformed grid voltages (1) with delay $\tau > 0$:

$$\begin{aligned} V_\alpha^\tau &= V_\alpha\left(t-\tau\right), \\ &= v_{\alpha 0} + v^+ \cos\left(\theta^+ - \omega\tau\right) + v^- \cos\left(\theta^- - \omega\tau\right), \end{aligned} \tag{11}$$
$$\begin{aligned} V_\alpha^{2\tau} &= V_\alpha\left(t-2\tau\right), \\ &= v_{\alpha 0} + v^+ \cos\left(\theta^+ - 2\omega\tau\right) + v^- \cos\left(\theta^- - 2\omega\tau\right), \end{aligned} \tag{12}$$
$$V_\beta^\tau = v_{\beta 0} + v^+ \sin\left(\theta^+ - \omega\tau\right) - v^- \sin\left(\theta^- - \omega\tau\right), \tag{13}$$
$$V_\beta^{2\tau} = v_{\beta 0} + v^+ \sin\left(\theta^+ - 2\omega\tau\right) - v^- \sin\left(\theta^- - 2\omega\tau\right). \tag{14}$$

From the Clark-transformed voltages and the consecutively delayed samples, one can find that:

$$V_\alpha - 2\cos\left(\omega\tau\right) V_\alpha^\tau + V_\alpha^{2\tau} = -2 v_{\alpha 0}\left(\cos\left(\omega\tau\right) - 1\right), \tag{15}$$
$$V_\beta - 2\cos\left(\omega\tau\right) V_\beta^\tau + V_\beta^{2\tau} = -2 v_{\beta 0}\left(\cos\left(\omega\tau\right) - 1\right). \tag{16}$$

Then, the DC offsets can be estimated as:

$$\hat{v}_{\alpha 0} = \frac{V_\alpha - 2\cos\left(\omega\tau\right) V_\alpha^\tau + V_\alpha^{2\tau}}{-2\left(\cos\left(\omega\tau\right) - 1\right)}, \tag{17}$$
$$\hat{v}_{\beta 0} = \frac{V_\beta - 2\cos\left(\omega\tau\right) V_\beta^\tau + V_\beta^{2\tau}}{-2\left(\cos\left(\omega\tau\right) - 1\right)}. \tag{18}$$

By subtracting the estimated DC offsets from the measured voltages, offset-free voltages can be obtained. Offset estimation equations (17) and (18) are frequency dependent. To avoid any coupling between the pre-loop DC estimation and the in-loop frequency

estimation, frequency-fixed implementation of (17) and (18) are preferable. This will necessitate in-loop compensation when the grid frequency varies from the nominal value. For this purpose, quantification of the frequency variation-induced error is required. This can be done by considering the transfer function of estimator in the phasor-form. Let us consider that $\tau = T_n/4$ with T_n being the nominal period. Then, transfer function of DC offset-free voltage estimator in the phasor-form can be obtained as:

$$G_{DC}(s) = \overrightarrow{\hat{V}}^{\emptyset}_{\alpha\beta}(s)/\overrightarrow{V}_{\alpha\beta}(s) = 0.5\left(1 - e^{-2\tau s}\right), \tag{19}$$

where $\hat{}$ and the superscript $\emptyset$ indicate DC offset-free estimation. Let us denote that $\omega = \omega_n + \tilde{\omega}$ with $\tilde{\omega}$ being the deviation from the nominal value. Then, the magnitude and phase of the transfer function (19) w.r.t. the frequency deviation can be obtained as:

$$|G_{DC}(j\omega)| = \cos(\tau\tilde{\omega}) \approx 1 - \frac{\tau^2}{2}\tilde{\omega}^2, \tag{20}$$

$$\angle G_{DC}(j\omega) = -\tau\tilde{\omega}. \tag{21}$$

In obtaining (20), small-angle approximation is used, i.e., $\cos(x) = 1 - x^2/2$. Equations (20) and (21) quantify the frequency deviation-induced errors when nominal frequency is used in estimating the DC offsets in (17) and (18). These errors need to be compensated in-loop to obtain error-free estimation of the signal parameters.

Implementation in a PLL

As shown previously, the proposed phase detector gives four DC signals that are related to the FPPS and FFNS components. From these DC signals, the uncompensated FFPS and FFNS amplitudes and FFPS phase angle can be estimated as:

$$\hat{v}^+ = \sqrt{\left(v_s^{f+}\right)^2 + \left(v_c^{f+}\right)^2}, \tag{22}$$

$$\hat{v}^- = \sqrt{\left(v_s^{f-}\right)^2 + \left(v_c^{f-}\right)^2}. \tag{23}$$

$$\hat{\phi}^+ = \text{atan2}\left(v_s^{f+}, v_c^{f+}\right). \tag{24}$$

The estimated FFPS phase angle can be considered as a mismatch between the input $\hat{\theta}$ and the output $\hat{\omega}t$. By passing this mismatch through a proportional controller with gain $\Omega > 0$, the frequency deviation and consequently the actual frequency can be estimated as:

$$\hat{\omega} = \Omega\hat{\phi}^+ + \omega_n, \tag{25}$$

where $\Omega > 0$ is the tuning gain. Then, using $\hat{\phi}^+$ and $\hat{\omega}$, the uncompensated instantaneous phase angle of the FFPS component can be estimated:

$$\hat{\theta}^+ = \hat{\omega}t + \hat{\phi}^+. \tag{26}$$

By taking into account the phase and amplitude error (*cf.* (20) and (21)) induced by the frequency-fixed operation of the pre-loop filter, the compensated amplitude and phase can angle can be found as:

$$\hat{v}_c^+ = \frac{\sqrt{\left(v_s^{f+}\right)^2 + \left(v_c^{f+}\right)^2}}{1 - \frac{\tau^2}{2}\tilde{\omega}^2}, \tag{27}$$

$$\hat{v}_c^- = \frac{\sqrt{\left(v_s^{f-}\right)^2 + \left(v_c^{f-}\right)^2}}{1 - \frac{\tau^2}{2}\tilde{\omega}^2}, \tag{28}$$

$$\hat{\theta}^+ = \hat{\omega}t + \hat{\phi}^+ + \tau\tilde{\omega}, \tag{29}$$

where the subscript c stands or compensated value.

A block diagram of the proposed technique is given in Fig. 1. In obtaining the DC signals (7)-(10), it has been assumed that the window length of MAF is equal to the nominal grid frequency period. However, in the presence of off-nominal harmonics and/or unbalance, small-amplitude oscillation in the estimated parameters are inevitable. In this case, frequency-adaptive MAF can be used and is considered in this work.

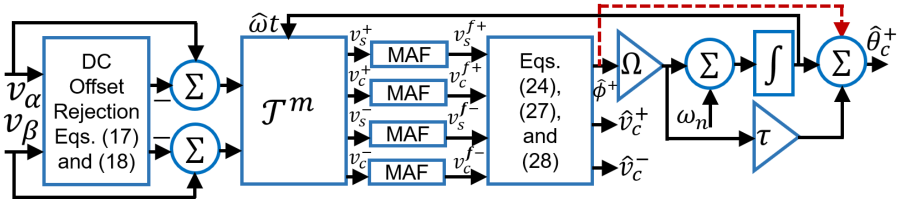

Fig 1. Block diagram of the proposed PLL.

Small-Signal Modeling

To develop the small-signal model of the proposed PLL, let us consider the block diagram of the proposed technique given in Fig. 1.

The FFPS phase angle can be written as $\phi^+ = \theta^+ - \hat{\omega}t$. As a result, the proposed phase angle detector takes θ^+ as the input and $\hat{\omega}t$ as the feedback signal. However, in the presence of unbalanced voltages, negative sequence AC components will appear which can be filtered out by MAF to extract the DC components. DC components V_s^{f+} and V_c^{f+} are passed by the arctangent block which linearizes the phase detector output. As a result, Fig. 2 can be considered as the small-signal model of the proposed technique where a synchronous reference frame version of the transfer function (19) [30, 40] is used. According to Fig. 2 , by using block diagram reduction algebra, the open-loop transfer function is given by:

$$G(s) = \frac{G_f(s)}{1 - G_f(s)\,(1 + \Omega\tau)} \left(\frac{\Omega}{s} + 1\right).$$

To validate the small-signal model, let us consider a $+15°$ phase change. Results of the simulation are given in Fig. 3. Based on the results in Fig. 3, it can be claimed that the small-signal model is very accurate.

Parameter Tuning

The proposed technique has one parameter to tune which is Ω. To tune this parameter, a settling time-based criterion is used. In this approach, +1Hz step change is considered and the corresponding 2% settling time is obtained. Settling time variation w.r.t. varying

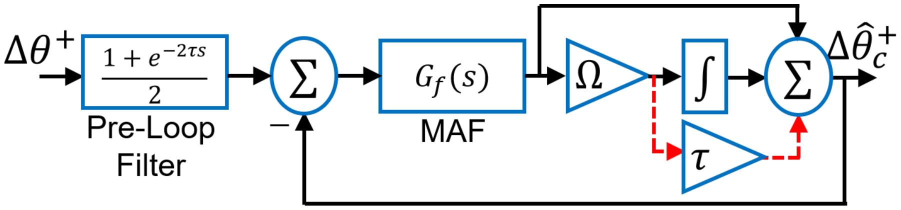

Fig 2. Small-signal model of the proposed technique.

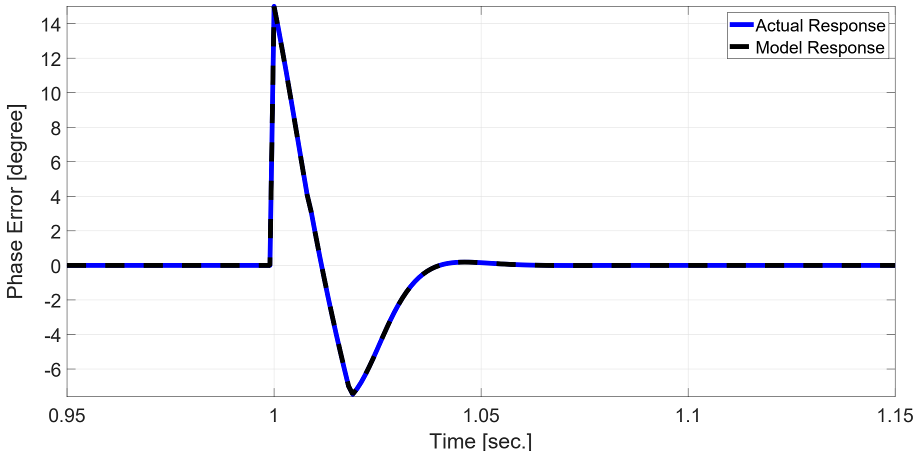

Fig 3. Small-signal model accuracy assessment for $+15°$ phase jump with $\Omega = 91$.

Ω and the corresponding open-loop phase margin are given in Fig. 4. From Fig. 4 it can be found that for $\Omega = 91$, the settling time has been found to be $\approx$ 30msec., which is the fastest convergence time. This value of Ω results in a phase margin of $\approx 35.5°$ which is within the recommended $30° - 60°$ range. As such, this value has been selected.

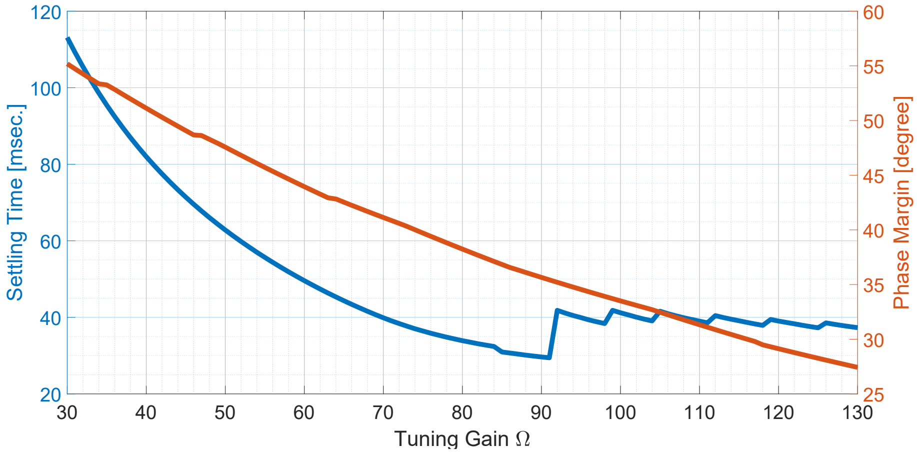

Fig 4. Parameter tuning plot for the proposed method.

Results and Discussion

In this Section, Matlab/Simulink-based numerical simulation and hardware-based experimental studies are considered. As comparative techniques, Cascaded delayed signal

cancellation (CDSC)-PLL [40] and quasi type-1 (QT1)-PLL [28] are selected. Control parameters are given in Table 1. For the sake of fair gain tuning, all the techniques are tuned using the same phase margin. The comparative techniques are implemented using a fixed-step solver with a sampling frequency of 10 kHz.

Table 1. Parameter values of the selected PLL techniques.

	QT1	CDSC	Proposed
Frequency Gain	71	99	91
MAF Window Length	T	$\frac{T}{4}$	$\frac{T}{2}$
DSC Window Length	–	$\frac{3T}{4}$	$\frac{T}{2}$
Phase Margin		$\approx 35.5°$	

Simulation Results

Balanced to Unbalanced and Distorted Test

In this test, initially the grid is balanced. Suddenly, the grid become unbalanced and distorted with $\overrightarrow{V}^{+1} = 0.733\angle 5°$ [p.u.], $\overrightarrow{V}^{-1} = 0.211\angle 50.4°$ [p.u.], $\overrightarrow{V}^{+5} = 0.054\angle 45°$ [p.u.], $\overrightarrow{V}^{+7} = 0.023\angle 60°$ [p.u.], and $\overrightarrow{V}^{+11} = 0.019\angle 90°$ [p.u.], 0.012 [p.u.] 20Hz sub-harmonics, and 0.009 [p.u.] 270Hz inter-harmonics. At the same time, grid frequency also experienced a jump of +1 Hz in frequency. Comparative numerical simulation results are given in Fig. 5. Despite the grid being heavily distorted, no steady-state oscillation in the estimated frequency can be observed for the proposed technique and the QT1. However, the same can not be said for CDSC. In calculating the settling time band, we have used the steady-state oscillation value of CDSC. As given in Table 2, the proposed method has roughly 50% faster convergence compared to the selected techniques despite tuned using the same open-loop gain. In this paper, estimation of the FFPS and FFNS component amplitudes are considered. The proposed technique estimated both sequence amplitudes in ≈ 1 cycle. This shows the suitability of the proposed method in monitoring grid unbalance.

Table 2. Comparative Time-Domain Simulation Summary of the Selected Techniques.

Attributes ⇓	QT1	CDSC	Proposed
Test-I: Balanced to Unbalanced Grid			
Frequency Settling Time (msec.)	40.2	40.2	24.7
Phase Settling Time (msec.)	43.5	41.5	21.4
Total Harmonic Distortion	0.29%	0.42%	0.29%
Test-II: Balanced to Unbalanced and Biased Grid			
Frequency Settling Time (msec.)	57.8	24.2	19.4
Phase Settling Time (msec.)	44.6	23.4	20.4

Unbalanced and Distorted to Balanced Test

In this test, initially the grid is unbalanced and distorted as per the condition specified in the previous test. Suddenly, the grid becomes balanced and the grid frequency returns to the nominal value. Comparative numerical simulation results are given in Fig. 6. The proposed technique can quickly detect the change in the grid and showed the fastest convergence speed in terms of frequency and phase estimation. Same as the previous test, the proposed technique estimated the FFPS and FFNS amplitudes in ≈ 1 cycle.

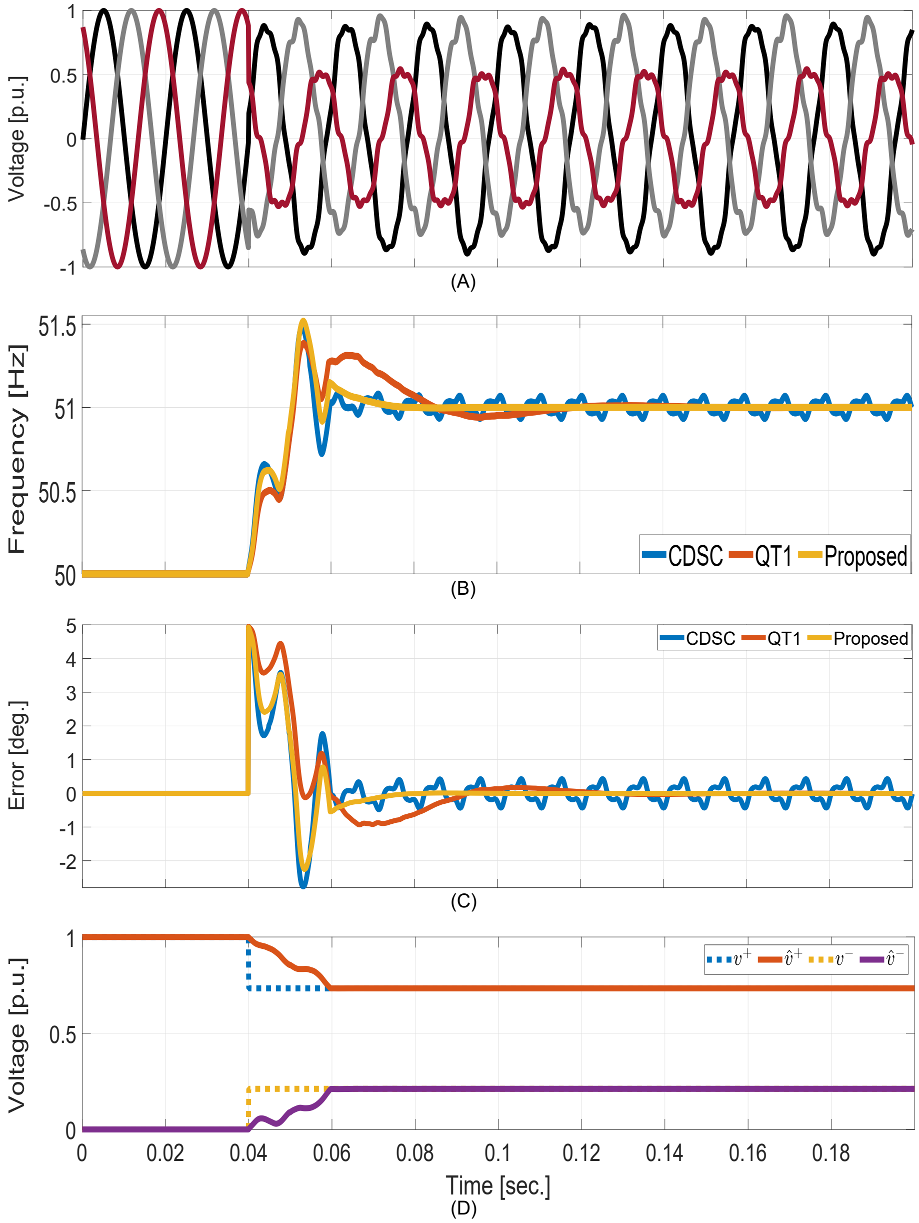

Fig 5. Comparative numerical simulation results for balanced to unbalanced and distorted test. A: Grid voltages. B: Estimated frequency. C: FFPS phase estimation error. D: Estimated FFPS and FFNS amplitudes .

A qualitative performance comparison of the selected techniques are given in Table As given in Table 3. Since the comparative techniques can not extract FFNS component, comparing the computational complexity of these techniques with the proposed method would be unfair. If only FFPS component estimation is considered, QT1-PLL [28] and the proposed technique have similar computational complexity. However, CDSC-PLL [40] has a higher computational effort as multiple DSC blocks are involved. Moreover, only certain sampling frequencies can be used to avoid fractional delay in implementing the DSC blocks. This also contributes to additional design complexity in selecting the filter components of the power converter as they are related to the sampling and switching

frequencies. This is not the case for the proposed method and the QT1-PLL. In addition, two Park transformation blocks are needed for the CDSC-PLL whereas QT1-PLL and the proposed method use one Park transformation. Despite having similar computational complexity, the proposed method has faster dynamic response compared to QT1-PLL. In addition, FFNS component estimation is another distinct feature of the proposed method compared to QT1-PLL and CDSC-PLL.

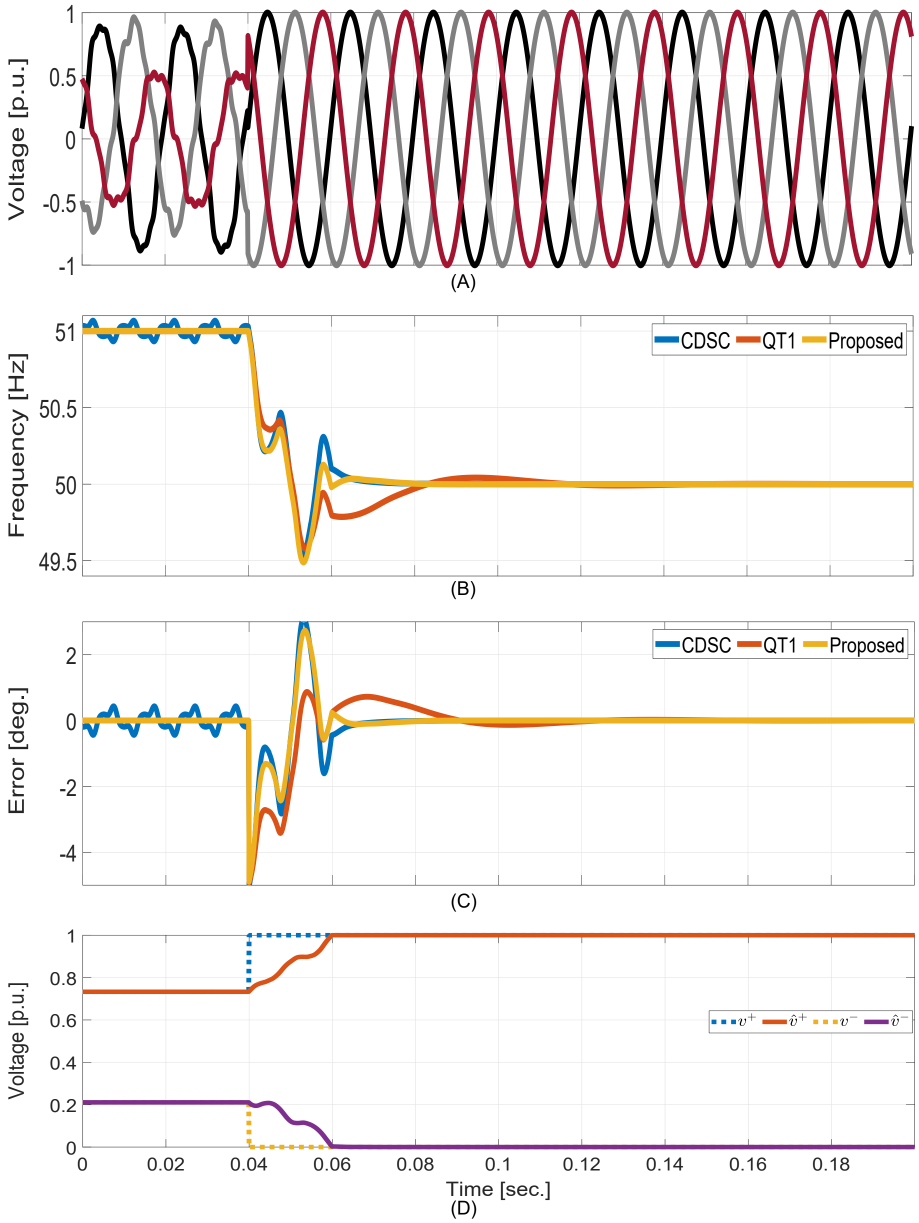

Fig 6. Comparative numerical simulation results for unbalanced and distorted to balanced test. A: Grid voltages. B: Estimated frequency. C: FFPS phase estimation error. D: Estimated FFPS and FFNS amplitudes .

Table 3. Qualitative performance comparison of selected PLLs.

Attributes ⇓	QT1	CDSC	Proposed
Dynamic Response	Good	Better	Best
Harmonic Robustness	Very Good	Good	Very Good
FFNS Amplitude Estimation	No	No	Yes

Experimental Results

The considered experimental test bench is shown in Fig. 7. Here, a PWM-controlled three-phase inverter is used to emulate the adverse grid voltage signal. The voltage sensors are used to measure the voltages at the load side. The control systems as well as the comparative techniques are implemented on a dSPACE 1104 real-time platform using 10 kHz sampling frequency. Technical details of the setup are given in Table 4. Results are exported to Matlab for plotting.

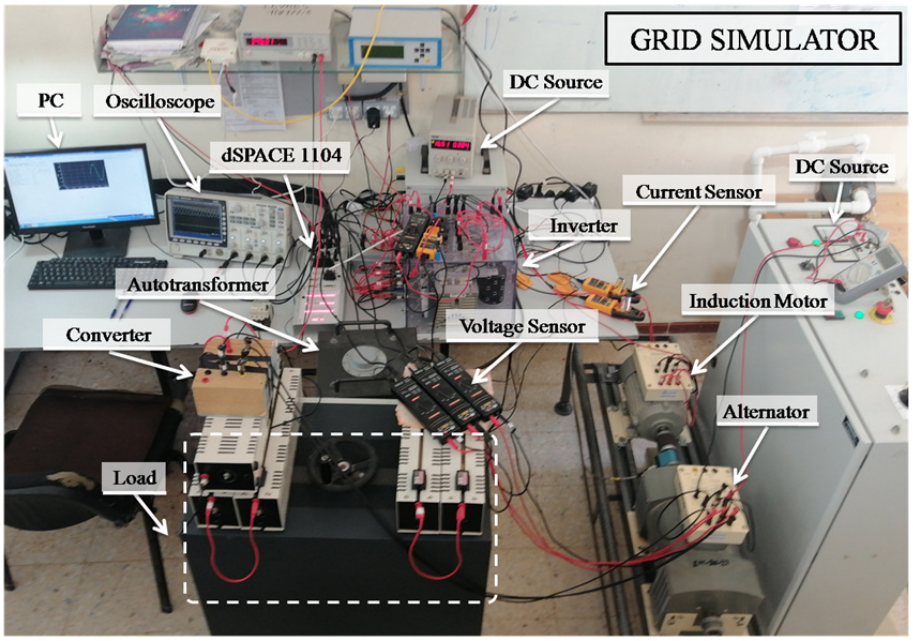

Fig 7. Test setup.

Table 4. System parameters.

Parameter	Value
DC-Link voltage	$V_{dc} = 310\,V$
Inverter voltage	$110\,V$ (rms)
LCL filter	Inverter and load side $L = 3$ mH, $C = 84\,\mu F$
Inverter rating	20 kVA
Frequency	Sampling and Switching: 10 kHz
Load parameter	$R = 167$ Ohm

To validate the theoretical development, three experimental tests are considered. In the first test, −2Hz frequency step is considered. From the comparative experimental results as shown in Fig. 8, one can find that both CDSC and the proposed method have roughly 1.5 cycles convergence time while it is two times higher for QT1. However, unlike CDSC, the proposed technique does not have any visible steady-state oscillation.

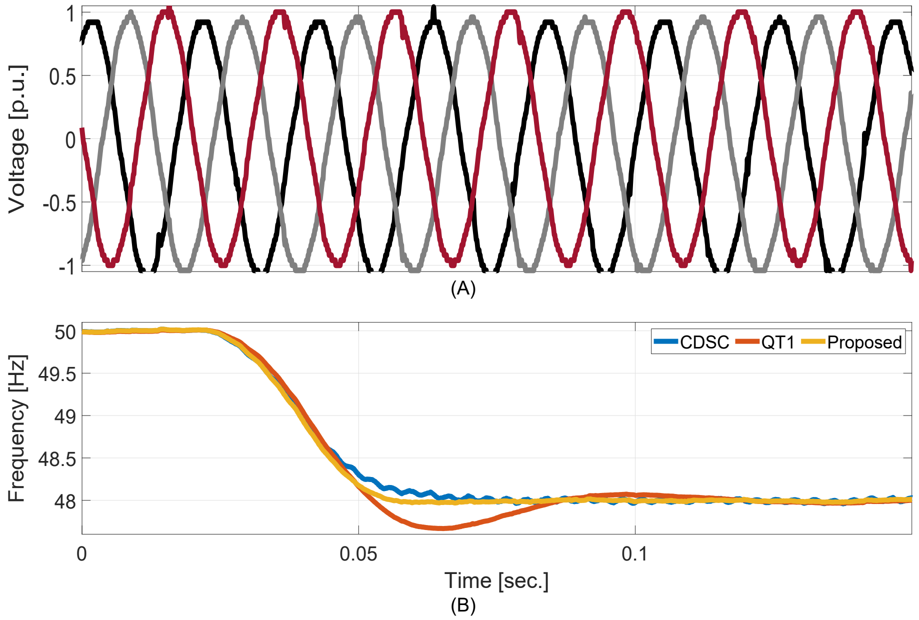

Fig 8. Comparative experimental results for -2Hz frequency jump. A: Grid voltage. B: Estimated frequencies.

In the second test, a large voltage sag is considered. Figure 9 shows the results for -0.5p.u. voltage sag. The proposed technique converged significantly faster than CDSC and QT1. Similar to simulation results, very fast convergence can be observed for the sequence amplitudes estimation.

In the final test, diode-rectifier nonlinear load is added to generate harmonics in the measured voltages. Moreover, the fundamental component amplitude also changed. Experimental results are shown in Fig. 10. They show that the proposed technique has a very high disturbance rejection capability. The estimated frequency contains almost no ripple despite the grid being highly distorted. It has faster convergence than QT1 and CDSC. This validates the excellent harmonics disturbance rejection capability of the proposed technique.

Conclusion

A grid-synchronization-based FFPS and FFNS sequence amplitudes estimator was proposed in this work. The sequence extractor relies on an enhanced phase detector that is inspired by the phase detector of the QT1-PLL. Moreover, it has a similar rotational speed as that of SRF-PLL. It can accurately extract the FFPS/FFNS phase-angles and based on that estimates the unknown grid frequency. It has a very good harmonic robustness property and can eliminate the effect of DC offset. Using various challenging test scenarios such as voltage unbalance, harmonics, DC offset, and frequency variation, the performance of the proposed technique was evaluated and compared with similar other MAF-based PLLs. Comparative results show that the proposed technique strikes a good balance between fast convergence and disturbance rejection capability. The proposed PLL is a type-2 control system. Further work is needed to make it type-3. This issue will be considered as future work.

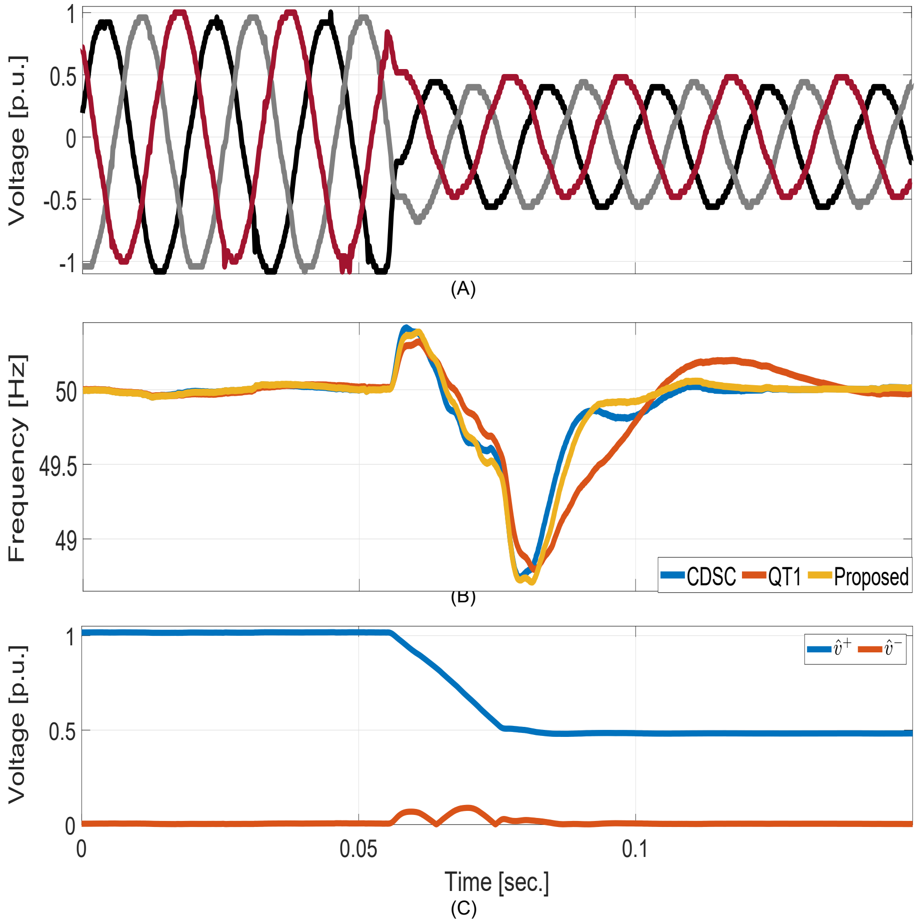

Fig 9. Comparative experimental results for voltage sag test. A: Grid voltages. B: Estimated frequencies. C: Estimated FFPS and FFNS amplitudes.

Supporting information

S1 File. Manuscript with Figures. This supplementary file contains the manuscript with all the figures in place.

References

1. Selvakumar RB, Vivekanandan C. Analysis of modified plug-in electric vehicle charger controller with grid support functionalities. PLOS ONE. 2022;17(1):e0262365. doi:10.1371/journal.pone.0262365.

2. Roslan MF, Al-Shetwi AQ, Hannan MA, Ker PJ, Zuhdi AWM. Particle swarm optimization algorithm-based PI inverter controller for a grid-connected PV system. PLOS ONE. 2020;15(12):e0243581. doi:10.1371/journal.pone.0243581.

3. Naderipour A, Zin AAM, Habibuddin MHB, Miveh MR, Guerrero JM. An improved synchronous reference frame current control strategy for a photovoltaic grid-connected inverter under unbalanced and nonlinear load conditions. PLOS ONE. 2017;12(2):e0164856. doi:10.1371/journal.pone.0164856.

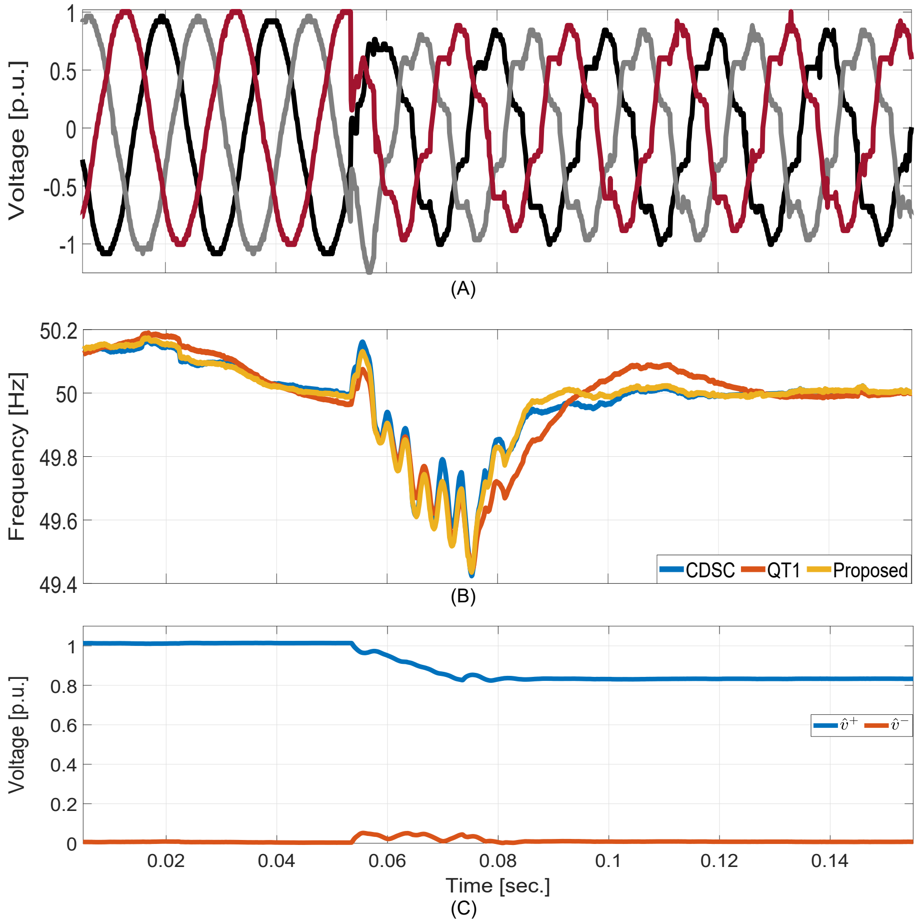

Fig 10. Comparative experimental results for nonlinear diode-rectifier load. A: Grid voltages. B: Estimated frequencies. C: Estimated FFPS and FFNS amplitudes.

4. Çelik D. Lyapunov based harmonic compensation and charging with three phase shunt active power filter in electrical vehicle applications. International Journal of Electrical Power & Energy Systems. 2022;136:107564.

5. Meral ME, Çelík D. A comprehensive survey on control strategies of distributed generation power systems under normal and abnormal conditions. Annual Reviews in Control. 2019;47:112–132.

6. Çelik D, Meral ME. Multi-objective control scheme for operation of parallel inverter-based microgrids during asymmetrical grid faults. IET Renewable Power Generation. 2020;14(13):2487–2498.

7. Albatsh FM, Ahmad S, Mekhilef S, Mokhlis H, Hassan MA. Optimal Placement of Unified Power Flow Controllers to Improve Dynamic Voltage Stability Using Power System Variable Based Voltage Stability Indices. PLOS ONE. 2015;10(4):e0123802. doi:10.1371/journal.pone.0123802.

8. Weidenbrug R, Dawson FP, Bonert R. New synchronization method for thyristor power converters to weak AC-systems. IEEE Transactions on Industrial Electronics. 1993;40(5):505–511.

9. Busarello TDC, Junior SLS, da Silva N. Zero-Crossing Detection Frequency Estimator Method Combined with a Kalman Filter for Non-ideal Power Grid. In: 2019 IEEE 15th Brazilian Power Electronics Conference and 5th IEEE Southern Power Electronics Conference (COBEP/SPEC). IEEE; 2019. p. 1–6.

10. McGrath BP, Holmes DG, Galloway JJH. Power converter line synchronization using a discrete Fourier transform (DFT) based on a variable sample rate. IEEE Transactions on Power Electronics. 2005;20(4):877–884.

11. Amirat Y, Oubrahim Z, Ahmed H, Benbouzid M, Wang T. Phasor Estimation for Grid Power Monitoring: Least Square vs Linear Kalman Filter. Energies. 2020;13(10):2456.

12. Roy S, Chishti F, Singh B, Panigrahi B. GNLMP Control for Solar PV-Battery Based Microgrid with Ms-EPLL Synchronization. In: 2021 IEEE 6th International Conference on Computing, Communication and Automation (ICCCA). IEEE; 2021. p. 988–994.

13. Ahmed H, Salgado I, Chairez I, Benbouzid M. Robust Gradient Estimator for Unknown Frequency Estimation in Noisy Environment: Application to Grid-Synchronization. IEEE Access. 2020;8:70693–70702.

14. Pay ML, Cao P, Sun Y, McCluskey D. Luenberger Observer Based Grid Synchronization Techniques for Smart Grid Application. In: IECON 2020 The 46th Annual Conference of the IEEE Industrial Electronics Society. IEEE; 2020. p. 4955–4960.

15. Kaura V, Blasko V. Operation of a phase locked loop system under distorted utility conditions. IEEE Transactions on Industry applications. 1997;33(1):58–63.

16. Meral ME, Çelik D. Benchmarking simulation and theory of various PLLs produce orthogonal signals under abnormal electric grid conditions. Electrical Engineering. 2018;100(3):1805–1817.

17. Rodríguez P, Pou J, Bergas J, Candela JI, Burgos RP, Boroyevich D. Decoupled double synchronous reference frame PLL for power converters control. IEEE Transactions on Power Electronics. 2007;22(2):584–592.

18. Chaoui H, Okoye O, Khayamy M. Grid synchronization phase-locked loop strategy for unbalance and harmonic distortion conditions. Journal of Control, Automation and Electrical Systems. 2016;27(4):463–471.

19. Biricik S, Redif S, Özerdem ÖC, Khadem SK, Basu M. Real-time control of shunt active power filter under distorted grid voltage and unbalanced load condition using self-tuning filter. IET Power Electronics. 2014;7(7):1895–1905.

20. Meral ME, Celik D. DSOGI-PLL based power control method to mitigate control errors under disturbances of grid connected hybrid renewable power systems. Advances in Electrical and Electronic Engineering. 2018;16(1):81–91.

21. Biricik S, Komurcugil H, Tuyen ND, Basu M. Protection of Sensitive Loads Using Sliding Mode Controlled Three-Phase DVR With Adaptive Notch Filter. IEEE Transactions on Industrial Electronics. 2018;66(7):5465–5475.

22. Mellouli MS, Hamouda M, Slama JBH, Al Haddad K. A third-order MAF Based QT1-PLL that is robust against harmonically distorted grid voltage with frequency deviation. IEEE Transactions on Energy Conversion. 2021;36(3):1600 – 1613.

23. Verma AK, Subramanian C, Jarial RK. An Error Demodulation Technique for Single-Phase Grid Synchronization/LVRT Applications. IEEE Systems Journal. 2021;doi:10.1109/JSYST.2021.3062878.

24. Mathuria K, Hussain I, Singh B, Kumar N. A quadrature oscillator-based DT for accurate estimation of fundamental load current for PV system in distribution network. IEEE Transactions on Industrial Informatics. 2018;15(6):3324–3333.

25. Ahmed H, Biricik S, Benbouzid M. Enhanced Frequency Adaptive Demodulation Technique For Grid-Connected Converters. IEEE Transactions on Industrial Electronics. 2021;68(11):11053 – 11062.

26. Safa A, Berkouk EM, Messlem Y, Chedjara Z, Gouichiche A. A pseudo open loop synchronization technique for heavily distorted grid voltage. Electric Power Systems Research. 2018;158:136–146.

27. Verma AK, Jarial RK, Roncero-Sanchez P, Ungarala MR, Guerrero JM. An improved hybrid pre-filtered open-loop algorithm for three-phase grid synchronization. IEEE Transactions on Industrial Electronics. 2021;68(3):2480 – 2490.

28. Golestan S, Freijedo FD, Vidal A, Guerrero JM, Doval-Gandoy J. A quasi-type-1 phase-locked loop structure. IEEE Transactions on Power Electronics. 2014;29(12):6264–6270.

29. Ahmed H, Biricik S, Komurcugil H, Benbouzid M. Enhanced quasi type-1 PLL-based multi-functional control of single-phase dynamic voltage restorer. Applied Sciences. 2021;12(1):146.

30. Sevilmiş F, Karaca H. A fast hybrid PLL with an adaptive all-pass filter under abnormal grid conditions. Electric Power Systems Research. 2020;184:106303.

31. Ahmed H, Tir Z, Verma AK, Elghali SB, Benbouzid M. Quasi Type-1 PLL With Tunable Phase Detector for Unbalanced and Distorted Three-Phase Grid. IEEE Transactions on Energy Conversion. 2022;37(2):1369–1378. doi:10.1109/TEC.2021.3130492.

32. Rodríguez P, Luna A, Candela I, Mujal R, Teodorescu R, Blaabjerg F. Multiresonant frequency-locked loop for grid synchronization of power converters under distorted grid conditions. IEEE Transactions on Industrial Electronics. 2011;58(1):127–138.

33. Golestan S, Ramezani M, Guerrero JM, Freijedo FD, Monfared M. Moving average filter based phase-locked loops: Performance analysis and design guidelines. IEEE Transactions on Power Electronics. 2013;29(6):2750–2763.

34. Roncero-Sanchez P, del Toro Garcia X, Torres AP, Feliu V. Fundamental positive- and negative-sequence estimator for grid synchronization under highly disturbed operating conditions. IEEE transactions on power electronics. 2012;28(8):3733–3746.

35. Miskovic V, Blasko V, Jahns TM, Harke MC. Resilient Synchronization of Grid Converters at Low Sampling Frequencies. In: 2020 IEEE Applied Power Electronics Conference and Exposition (APEC); 2020. p. 753–760.

36. Karimi-Ghartemani M, Khajehoddin SA, Jain PK, Bakhshai A, Mojiri M. Addressing DC component in PLL and notch filter algorithms. IEEE Transactions on Power Electronics. 2011;27(1):78–86.

37. Kherbachi A, Chouder A, Bendib A, Kara K, Barkat S. Enhanced structure of second-order generalized integrator frequency-locked loop suitable for DC-offset rejection in single-phase systems. Electric Power Systems Research. 2019;170:348–357.

38. Ahmed H, Ushirobira R, Efimov D. A Simple Frequency Estimator for Power Systems. IEEE Transactions on Instrumentation and Measurement. 2021;70:1–2. doi:10.1109/TIM.2021.3094249.

39. Ahmed H, Ushirobira R, Efimov D. On Biased Harmonic Signal Estimation: Application to Electric Power Grid Monitoring. IEEE Transactions on Control Systems Technology. 2022; p. 1–8. doi:10.1109/TCST.2022.3155322.

40. Sevilmiş F, Karaca H. Implementation of enhanced non-adaptive cascaded DSC-PLLs for renewable energy systems. International Journal of Electrical Power & Energy Systems. 2022;134:107470.
